# Predicting the effectiveness of interventions on population‐level sodium reduction: A simulation modeling study

**DOI:** 10.1002/hsr2.540

**Published:** 2022-03-07

**Authors:** Yiping Zeng, Zeshui Xu, Yu Rao

**Affiliations:** ^1^ Department of Management Science and Engineering Business School, Sichuan University Chengdu China; ^2^ Department of Bioengineering Food Science and Bioengineering School, Xihua University Chengdu China

**Keywords:** cancer burden, China, dietary‐sodium, health disparities, interventions

## Abstract

**Background and Aims:**

Interventions that significantly reduce dietary sodium intake are anticipated to decrease gastric cancer (GCa) burden. However, the optimal restriction strategies remain unknown at present. This study aims to understand where and to what extent policies modifying sodium consumption change the distribution of GCa burden, and the effects of potential salt reduction strategies in China.

**Methods:**

The synthetic population in this microscopic simulation study is close to reality. We incorporated estimates of dietary patterns and GCa risk into the model of excess salt consumption. These estimates and simulated population were obtained from the China Health and Nutrition Survey, Global Burden of Disease Project, and the sixth census of China's National Bureau of Statistics, respectively.

**Results:**

In the no intervention scenario, we estimated that disease burdens due to excess sodium intake would be at 472.9 million disability‐adjusted life years (DALYs) nationally between 2010 and 2030 (95% credible interval [CrI]: 371.1–567.7). The GCa burden caused by high sodium is projected to have a disproportionate impact on the central and southern provinces of China (9.2 and 4.5 million DALYs, respectively). Implementing a cooking salt substitute strategy would be expected to avoid a larger portion of GCa burden (about 67.2%, 95% CrI: 66.8%–67.6%) than the salt‐restriction spoon program (about 16.7%, 95% CrI: 16.1%–17.4%).

**Conclusion:**

Dietary salt reduction policy is very powerfully effective in reducing the GCa burden overall. It is expected that proposed salt substitutes are more effective than traditional salt‐restriction spoons to avoid increased inequality.

## 1 INTRODUCTION

China's National Nutrition Plan (2017–2030)^1^ objectives include reducing the national average daily salt intake by at least 20%. Although the high sodium diet is associated with an increased risk of gastric cancer (GCa),^2^, ^3^ adults in China, particularly those living in the central region, consume far more than the recommended amount in Chinese Dietary Guidelines.^4^ Since the majority of sodium intake has come from the consumers' additional table salt during cooking,^5^, ^6^ which accounted for 67% of the total sodium intake, an effective strategy for the sodium intake reduction has not been to reformulate packaged and prepared foods,^7^ as in the United States^8^ and other developed countries.^9^ To date, China has not formulated any sodium reduction strategy nationwide.^10^ Studies in Chinese participants suggest two dietary salt reduction strategies special for China: salt‐restriction spoons and salt substitutes.^11^, ^12^, ^13^ The obvious diversity of dietary sodium intake exists among different geographic locations, gender, or age group in China. However, it is still unclear where and to what degree change sodium intake may increase inequality in GCa burden, and which interventions may best mitigate the increase.

Previous mathematical modeling studies have proposed that considerable reductions in sodium intake would be expected to decrease cardiovascular disease^14^ and GCa.^15^, ^16^ Previous studies have examined the potential impact of dietary salt restriction strategies in adults over the age of 35 and particularly among hypertensive persons, but epidemiological evidence also shows an association between the longer‐term dietary and eating behavior in childhood or adolescence and dietary pattern in adulthood in some studies.^17^, ^18^ Some successful salt reduction strategies, such as public awareness campaigns, food labeling, and reformulation of processed foods, have been implemented in select cities, counties, and even provinces in China. However, these interventions have not been systematically assessed at interprovincial and interregional. Yet three questions remain unsettled. The first question is whether the potential interventions could produce effect through changes in dietary patterns of various age groups. Diseases caused by excessive sodium intake among young adults under 30 are not as common as in the elderly, however, the prevalence of GCa has remained stable or even slightly increasing trend, and which restriction strategies may best mitigate the increase.^19^ Second, how much effect would be expected if the salt reduction strategy currently being implemented in individual provinces of China were extended to more regions. The third unresolved question is whether the salt reduction policy can reduce the inequality of the GCa burden caused by the difference in sodium intake among provinces and regions. We sought to address these questions using individual‐level data from health surveys, to examine the potential implications of interventions aimed at reducing salt consumption on reducing health inequalities ideally.

We developed a microsimulation model of salt consumption to project the health effects of GCa based on the disability‐adjusted life years accumulated over the period 2010–2030. We utilized this model to evaluate which strategy may best reduce the burden of GCa due to excess salt consumption. Specifically, we used a detailed table of the sodium content of foods and linked this information to 24‐h dietary recalls from a Health and Nutrition Survey sample of 22 887 people; ensure that the age and sex of the synthetic individuals are similar to that of the China population in end‐2010; we estimated the extent of sodium reduction and also quantified GCa burden in disability‐adjusted life years (DALYs).

## 2 METHODS

**Figure 1:**
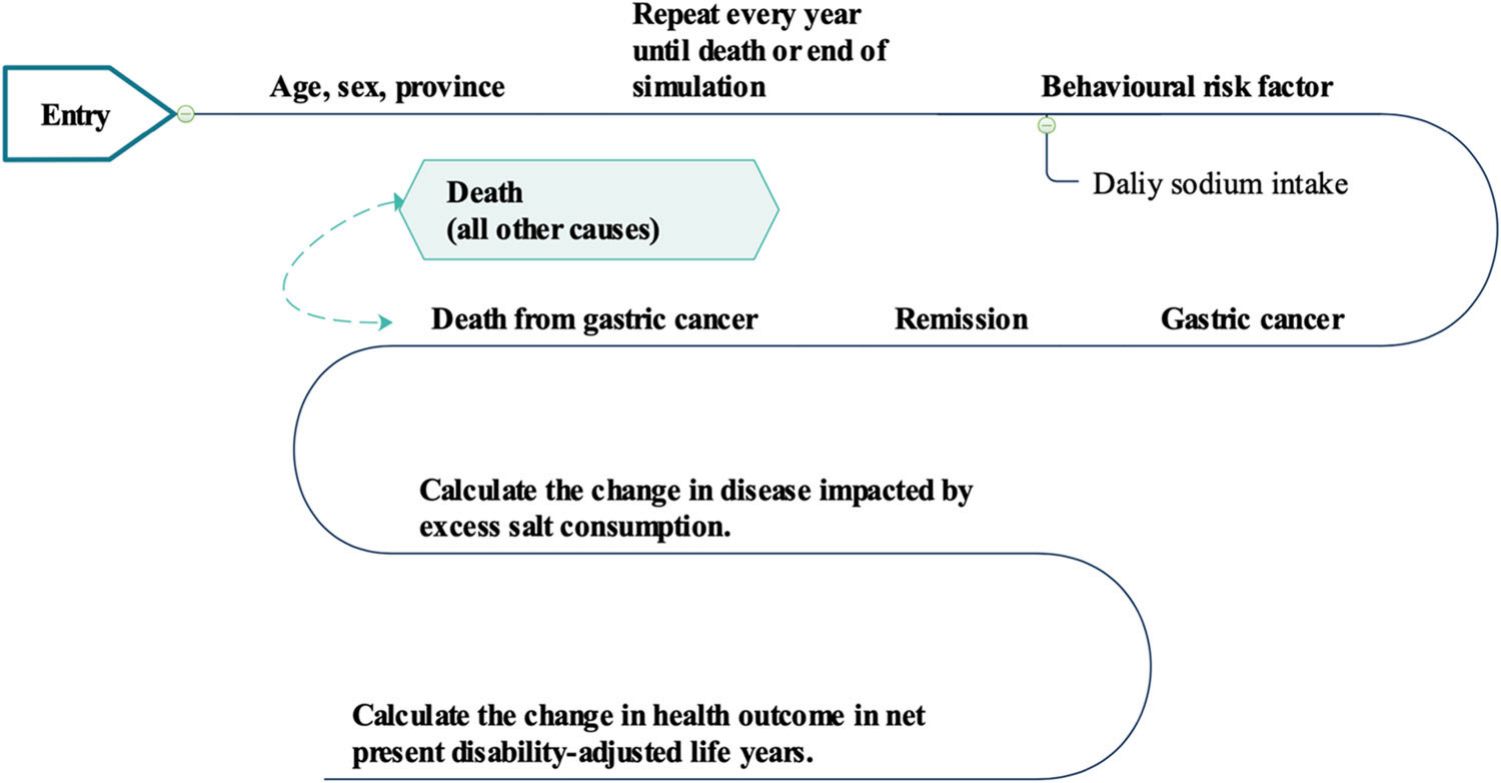
Model schematic. Data on salt consumption are used to calculate the change in sodium intake distributions and as inputs for microsimulation to project health outcomes due to sodium changes

We constructed a discrete‐time stochastic microsimulation model (Figure 1) to estimate the impact and equality of China‐specific cooking salt reduction interventions on GCa burdens. The model synthesizes information from the National Bureau of Statistics (NBS)^20^ regarding China population structure by province and the Health and Nutrition Survey for China (CHNS: 2004–2011)^21^ regarding sodium intake estimation procedures to generate a close‐to‐reality simulated population.^22^ This model simulates synthetic individuals rather than aggregate population averages (i.e., a Markov cohort model) and reflects the range of sodium intake given the disparities in dietary patterns in each province, and associated sodium excess status and attendant risk for GCa.

### 2.1 Simulated population

We simulated 10 000 individuals resembling the 2010 China population to match province‐specific gender, age (0–1 year, 1–4 year, and then by 5‐year increments), and death trends. We evaluated all provinces subject to CHNS data availability (Supporting Information Text S1). We calculated the results of each region based on geographic location and major dietary differences, including north (Beijing, Heilongjiang, and Liaoning), central (Henan, Jiangsu, and Shandong), and south (Chongqing, Hunan, Hubei, Guizhou, Guangxi, and Shanghai). As the simulation progressed in annual circles, the model included five potential actions for an individual: (1) updating risk factors (age, sex, and sodium intake), (2) updating sodium excess consumption statuses, (3) updating GCa statuses, (4) deaths, and (5) applying interventions. Each simulated individual was given probabilistic values for sodium consumption, GCa incidence risk, and mortality risk. The probability distributions of daily sodium intake were based on 24‐h dietary recalls data of CHNS, and modified for within‐person variations in consumption to estimate usual daily intake.^23^ Table 1 highlights key model components. See Figure S1 for a map of modeled provinces.

**Table 1:** Model parameters and sources

Parameters	Source
Population size of demographic cohorts	The Sixth National Census of China^20^ (National Bureau of Statistics 2010)
Sodium intake	China Health and Nutrition Survey^21^ ^,^ ^a^ (CHNS: 2004–2011)
All‐cause mortality rate	
	GBD^24^ ^,^ ^a^
Gastric cancer mortality rate	GBD^24^ ^,^ ^a^
Gastric cancer prevalence	GBD/WHO^24^ ^,^ ^25^ ^,^ ^a^
Gastric cancer disability weights	GBD^26^
RR gastric cancer given diet high sodium	GBD^26^

Abbreviation: RR, relative risk.

^a^ Calculated value.

### 2.2 Risk factor modeling and model outputs

The focus of this study was primary prevention, hence only the first episode GCa was considered. Trends of GCa‐associated risk factors were considered by projecting trends observed in CHNS from 2004 to 2011 (Supporting Information Text S1). The exposure of simulates individuals to sodium was informed by four health and nutrition surveys employing a combination of weighing and three consecutive 24‐h recalls between 2004 and 2011.^23^ The four dietary surveys were performed in the years 2004, 2006, 2009, and 2011 (Tables S1 and S2). In the nine provinces (including Guangxi, Guizhou, Heilongjiang, Hubei, Hunan, Henan, Jiangsu, Liaoning, and Shandong) covered by CHNS, we simulated the decline in sodium intake observed between 2004 and 2011, assuming a logarithmic decline. In the three megacities newly added to the CHNS in 2011 (Beijing, Chongqing, and Shanghai), we assumed that when no salt‐related interventions were implemented sodium exposure remained stable at the estimated level of 2011 until the period up to 2030.

The main source of dietary sodium in China was direct addition, followed by soy sauce, processed foods, and monosodium glutamate (MSG). To estimate the impact of interventions on total daily per capita sodium intake (i.e., sodium from all foods and condiments, including direct addition, soy sauce, and MSG) of each simulated individual, we used the Chinese food‐composition table (FCT) food^27^ codes and descriptions match food items contained in each 24‐h dietary recall in CHNS. The ideal level of sodium consumption has been unclear and heavily debated (appendix text S4 in Mozaffarian et al).^28^ We allow the risk‐free optimal sodium intake to be 0.6–2 g/day, and the model is 1.5 g/day, following the WHO recommended reference intake,^29^ a PERT distribution^30^ and the newly released US DRIs.^31^

Given the annual sodium intake level of each simulated individual, we determined whether an individual was above the ideal sodium intake level that would be marked “excess” and would make the individual vulnerable to disease. Excess salt consumption is associated with a heightened relative risk (RR) of morbidity and mortality from GCa among young adults aged 18–30, and there is a lag between exposure and GCa.^26^ In the simulation, we assumed that a direct effect through excess sodium consumption on GCa incidence with a mean lag time of 8 years.^32^ Due to the assumed lag times, any changes in sodium exposure between 2004 and 2011 will reflect the morbidity and mortality of GCa over the period 2012–2019. Health status in the model included healthy, sodium excess, GCa, and dead. During each loop, a person could only be in one set of health states. Health states for each person were assigned at the start of the model and after each loop based on the risk factor. Deaths were stochastically determined by a binomial probability function based on mortality rates for a person's demographic group and sodium excess status.

Demographic‐specific life expectancies and mortality rates were estimated from World Health Organization (WHO).^25^ Prevalence rates, mortality rates, and trends over time for GCa were obtained from the Global Burden of Disease Project (GBD).^24^ The prevalence and mortality trends of GCa assumed that the annual percentage changes in per capita mortality and prevalence rate from 1990 to 2008 obtained from GBD data were continuous. The model calculated disease burden in net present DALYs, which are the sum of years lived with disability and years of life lost, discounted at an annual rate of 3%. The disability weight of GCa was obtained from the GBD.^26^ In principle, disability weights might vary by age, gender, and personal preference. However, the GBD reports disability weight that is not stratified by these factors. Thus, in keeping with the disability weight methodology set by the GBD, our model did not use stratified disability weights. The primary microsimulation model outcome was the total morbidity and mortality burden associated with excess sodium intake per 1000 people and the overall synthetic population.

We considered culturally tailored dietary salt restriction strategies to mitigate the disease burden from excess sodium consumption. Specifically, we compared two salt reduction strategies to keep the daily sodium intake within the ideal state. The two public health strategies were (i) restrictions on added salt, including a series of interventions (e.g., promote the use of 2‐g salt spoons, teach the measurement of cooking salt, and educate on the potentially harmful effects of excess salt consumption)^11^; (ii) salt substitutes, in which is composed of 65% sodium chloride (NaCl), 25% potassium chloride, and 10% magnesium sulfate.^12^, ^13^ In the first strategy, we used 2‐g salt spoons to control the addition of salt during food preparation. In the second strategy, we simulated scenarios that only a certain portion of the five types of high‐sodium foods (added salt, soy sauce, MSG, fermented products, and pickled foods) were replaced by low‐sodium salts rich in potassium and magnesium.

### 2.3 Model validation

Evaluate the validity of model estimates by comparing model estimates with values reported in the literature.^33^, ^34^, ^35^ For risk factor trends validation, modeled mean sodium intake in 2015 was compared to the estimated exposure from CHNS. For external verification of disease burden, modeled deaths, DALYs from GCa from 2010 or 2010 to 2030 were compared to estimates from the GBD. Methods of calculating GBD country‐level aggregate estimates used for validation were not involved simulation modeling predictions but rather contemporaneous Bayesian statistics (Supporting Information Text S1).

### 2.4 Sensitivity analyses

First, we performed one‐way sensitivity analysis in which parameters change by 10% at a time in all model inputs (Table S3). In specially, we varied the initial sodium intake distribution across the range to account for potential changes in dietary patterns and the uncertainty of current measurement quality. Next, we evaluated how constant disease prevalence rates would affect model results, and assumed that GCa prevalence rate in 2010 would continue to exist during the model period. Finally, we performed probabilistic sensitivity analysis to test the robustness of the projected disease burden to changes in combinations of inputs. The model was run 10 000 times, while Monte Carlo sampling was taken from each probability distribution of each input parameter to calculate the uncertainty interval for each outcome. The model was programmed in R version 3.5.0 (R Project for Statistical Computing).^36^

## 3 RESULTS

### 3.1 Model validation

For this study, we calibrated the model to observed historical trends in excess salt consumption from 2004 to 2011 for each subpopulation. Our model‐based estimates of sodium intake and GCa were highly consistent with current estimates from other sources (Tables S4–S6). Specifically, our estimates of sodium intake in the three regions were within 1.2%, 0.1%, 7.7%, respectively, relative error from values in the literature in 2015.^33^, ^34^ Our estimate of the 2010 DALY burdens due to GCa was within 1.9% of value in the literature.^35^ It was estimated that DALY burdens due to GCa from 2010 to 2030 was within −0.8% of the literature value. All other model‐based results showed a high degree of agreement with prior estimates of current excess salt consumption and GCa.

### 3.2 Disease burden measures

According to our model, excess salt consumption would be expected to induce 472.9 million DALYs without any interventions in China over the period 2010 to 2030 (95% credible interval [CrI]: 371.1–567.7). If the relative burden caused by other risk factors did not change in China, this burden would account for approximately 13% (=472.9/3754 million DALYs) of the expected DALYs due to all diseases of any type during this period. The effect was that a high‐sodium diet would be the third leading modifiable risk factor for DALYs.^37^

**Figure 2:**
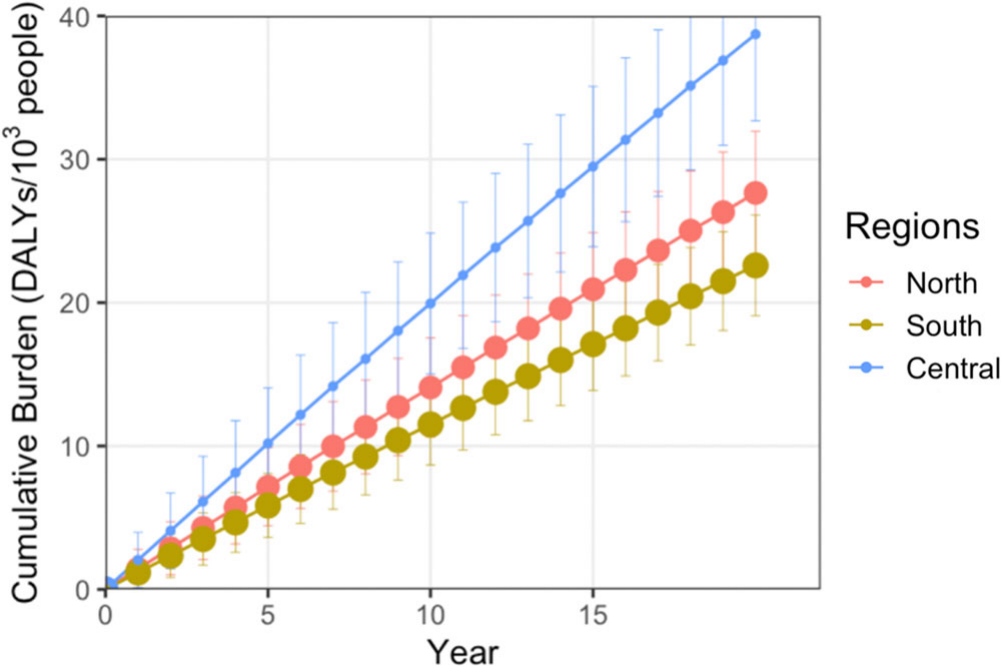
Cumulative per capita burden of gastric cancer (GCa) due to excess salt consumption in the scenario without interventions, 2010–2030. These points show average cumulative DALYs per 1000 population, and the error bars reflect a simulation of the probability that 10,000 people are above and below the average by one standard deviation

The model results suggested that excess salt consumption would increase the interprovincial and interregional inequality of China's per capita DALY burdens (Figure 2, Table S10). The region with the highest initial per capita burdens caused by the high‐sodium diets would be expected to be the most affected during the 20‐years, and the disparities would be increased over time. For example, during the study period, the per capita burden in North China would be estimated to be about 1.2 times that in the South. In addition, the central region with the highest per capita burdens due to the use of salt and large amounts of soy sauce during preparation cuisines (e.g., Shandong, Henan) are currently the regions with the highest estimated burdens due to excess salt consumption.

### 3.3 Restriction strategies

Compared with the salt‐restriction spoons intervention, salt substitutes would be expected to avoid the largest part of the cumulative projected burden caused by diet in high sodium over the period 2010–2030 (Tables 2, 3). Even with the use of 2‐g free salt spoons and with intensive added salt reduction strategies for related diseases, the sum of the cooking salt‐restriction strategy would be anticipated to reduce only 16.7% of all GCa caused by excess salt consumption (95% CrI: 16.1%–17.4%). We estimated that salt substitutes would be expected to avoid 67.2% of the GCa burden caused by excess salt consumption (95% CrI: 66.8%–67.6%).

**Table 2:** Effect of restriction strategies, 2010–2030

	Reduction in cumulative burden (%)
Salt‐restriction spoon addition	
1/4 salt‐restriction spoon (197 mg)	20.42 (=3.71/18.17)
1/2 salt‐restriction spoon (394 mg)	19.54 (=3.55/18.17)
3/4 salt‐restriction spoon (591 mg)	17.34 (=3.15/18.17)
1 salt‐restriction spoon (788 mg)	16.73 (=3.04/18.17)
Percentage of consuming highest sodium foods	
95% substitution	66.43 (=12.07/18.17)
85% substitution	65.44 (=11.89/18.17)
75% substitution	65.00 (=11.81/18.17)
65% substitution	64.61 (=11.74/18.17)

*Note*: Values are percent (the actual numerator and denominator). Results are shown that the percent reductions in cumulative disability‐adjusted life year burdens (DALYs) due to the decline in salt consumption caused by intervention from 2010 to 2030.

**Table 3:** Select intervention results by provinces

Province	Salt addition (%)	Salt substitutes (%)
Beijing	14.03 (=2.55/18.17)	67.12 (=12.20/18.17)
Liaoning	18.22 (=3.31/18.17)	66.83 (=12.14/18.17)
Heilongjiang	18.27 (=3.32/18.17)	67.95 (=12.35/18.17)
Shanghai	12.16 (=2.21/18.17)	66.71 (=12.12/18.17)
Jiangsu	16.40 (=2.98/18.17)	66.54 (=12.09/18.17)
Shandong	18.99 (=3.45/18.17)	67.02 (=12.18/18.17)
Henan	19.04 (=3.46/18.17)	68.25 (=12.40/18.17)
Hubei	18.01 (=3.27/18.17)	67.19 (=12.22/18.17)
Hunan	15.96 (=2.90/18.17)	66.89 (=12.15/18.17)
Guangxi	15.82 (=2.87/18.17)	67.69 (=12.30/18.17)
Guizhou	17.01 (=3.09/18.17)	67.07 (=12.19/18.17)
Chongqing	16.57 (=3.01/18.17)	67.15 (=12.21/18.17)

*Note*: Values are percent (the actual numerator and denominator). The mean percent reductions in cumulative disability‐adjusted life year burdens (DALYs) due to the decline in salt consumption caused by intervention from 2010 to 2030 are shown.

**Figure 3:**
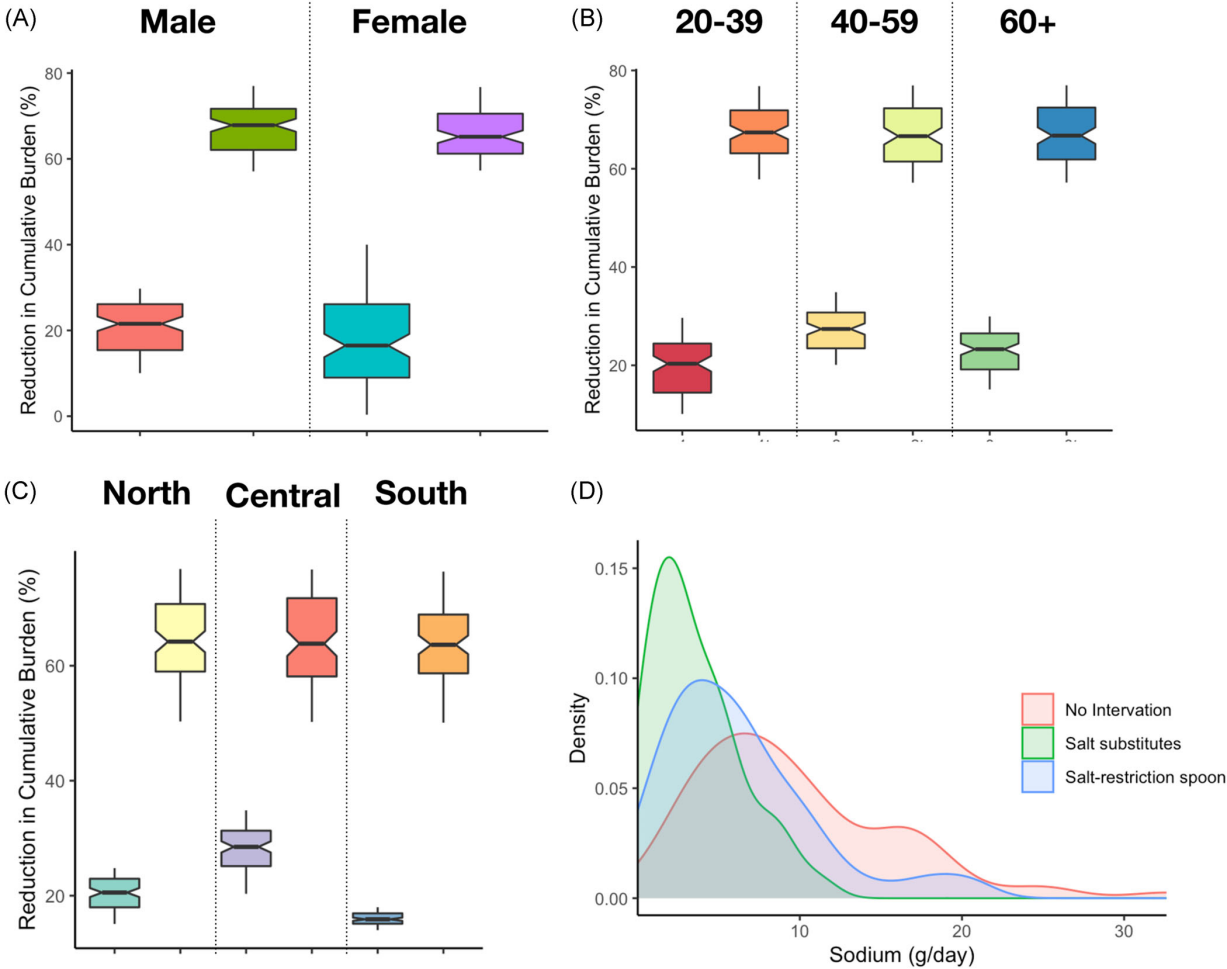
The percentage change in the cumulative burden of salt restriction spoons (left) and salt substitution (right) is calculated based on demographic characteristics, respectively: (A) sex, (B) age, (C) region. (D) shows the estimated daily sodium intake by an iterative simulation before and after the intervention

The provinces that benefited most from the salt reduction strategy were those with higher DALY burdens of current excess salt consumption (e.g., provinces in China's central region), because those provinces are expected to experience long‐term excess salt consumption and the largest number of new GCa cases over the study period. It is worth noting that in the modeled provinces, although there are considerable changes in the GCa burden induced by high sodium between iterations (Figure 2), the reduction in cumulative burden is relatively small (Table 3). We estimate that individuals in all population cohorts would benefit from dietary sodium reductions in the implementation of interventions (Figure 3). The reductions in cumulative burden would be expected to be largest among adults in the 40–59 age group. The percentage of adults over the age of 60 in the cumulative burden changed slightly but not significantly compared to the 40–59 age group.

### 3.4 Sensitivity analyses

By using a wide range of literature‐based alternative input parameters, the relative advantages of salt substitutes and salt‐restriction spoons were unaltered (Tables 2, 3, and S7–S9). Specifically, the relative superiority of salt substitutes did not change qualitatively with alterations in baseline sodium intake, GCa prevalence, or mortality.

## 4 DISCUSSION

We present an analysis of the impact of China's potential salt reduction policies on the burdens of GCa by simulating individuals. DALYs caused by excess sodium intake are anticipated to disproportionately affect provinces with high existing burdens, by our model, thereby increasing the inequality of the existing burdens of related diseases in China in the scenario without interventions. Compared to previous studies, we modeled specific potential interventions in China using a microsimulation model to project the GCa burden due to the high sodium intake. The model synthesizes information from the best available sources of information about the population's exposure to salt to generate a synthetic population close to reality. Critically, this method allowed us to understand the important health effects of changes in sodium intake in the context of the existing GCa burdens and health inequalities between provinces and regions.

While strategies to reduce sodium intake are different from those adopted in the Western world, consistent with the positive impact of their national salt reduction programs,^38^ our findings suggest that expansion of interventions would be likely to significantly reduce the GCa burden in china. This finding suggests that salt substitutes would be expected to avoid most (about 67.2%) of the projected increase in GCa caused by excess sodium intake, which is about three times greater than the use of salt restriction spoons to counteract the heightened risk of GCa. A 67.2% reduction in DALY burden due to high sodium intake is reasonable, as the intervention reduces the salt consumption by approximately 67.2% in 2030, and a linear dose‐response relation between salt consumption and risk of GCa was assumed. Yet this linear relationship need not be necessarily the case, nor is it a noticeable result, as relationships between excessive sodium intake, gastric cancer, and the resulting DALY burdens might create potential nonlinearities. Although added dietary salt is still the main source of sodium intake in China, with the change of lifestyle and dietary pattern caused by economic development, the opportunity to salt added during cooking has been reduced to varying degrees.^39^ For example, salt used to be a major food preservative in China, but with modernization and urbanization, the need to use it to preserve food has been greatly reduced. In addition, refrigeration and larger purchases of packaged processed foods have contributed to a marked shift away from traditional homemade high‐sodium pickled foods. Salt substitutes were more effective than the salt‐restriction spoon program because it benefited the entire population. Salt‐restriction spoons targeted the population with high levels of salt consumption, ignoring substantial population with a significant decline of salt consumption but still higher than the ideal level. Furthermore, salt substitutes reversed excess salt consumption‐induced related diseases, unlike using the 2‐g salt spoons, which addressed only a single form of reducing sodium intake. Although low‐sodium salts as a restriction strategy for large‐scale population‐wide are more beneficial in reducing sodium intake, caution should be exercised in the application of precautionary principles. A medicine institute committee found limited evidence that excessively low sodium may be associated with heightened mortality in older female adults and some patients with congestive heart failure, diabetes, or chronic kidney disease.^40^, ^41^, ^42^ Hence, interventions should be implemented with consideration in minimizing adverse effects on the most vulnerable individuals.

The overall health potential from salt restriction strategies is likely to be great, however, the benefits and potential risks may be heterogeneous among the different populations. The provinces in central China with the highest existing health burden would be expected to benefit most, while people in the south with near‐ideal intakes may be subjected to higher risks from very low sodium levels. Considering the food choice preferences in the South, as well as more health‐conscious participation in awareness campaigns and attention to food labels, salt substitution is significantly more effective than limiting salt spoons. Furthermore, the huge differences in cumulative burden among provinces in various regions may be due to socioeconomic inequality.^43^ The projected benefits of reducing the GCa burden would be expected to be largest among adults aged 40–59 years given their food consumption profiles and the expected reduction in sodium content in each food based on salt substitution targets. The present rate of reduction in the cumulative burden among these expected to benefit from interventions suggests a limited health effect for salt restriction strategies targeting adults only.^44^ Due to the long‐term unchanged diet and consumption of less food for adults over 60, the interventions did not significantly reduce the cumulative burden. Our study suggests that careful consideration should be given to how to address such large‐scale population‐wide sodium reduction strategies to avert the increased health inequalities due to dietary in high sodium.

We bring together the best available epidemiological data, methods, and the way consumers behave in terms of current food preferences across all age groups to calculate expected cumulative burden changes, enhancing the findings from previous sodium reduction models in China. Our model uses a 20‐year projection because this is the duration during which the uncertainty in the model remains manageable. In addition, we conduct extensive sensitivity analysis to test the assumptions made when developing the model and understand the impact of parameters with a large amount of uncertainty. Our analysis has many limitations, three of which are noteworthy. First, the model projects change in sodium intake based on the implementation of interventions to predict the resulting changes in disease burden. There is evidence to suggest that sodium intakes are underestimated in recent survey years, and there remains substantial uncertainty around what variations in dietary patterns in the long term with sustained implementation of interventions.^39^, ^45^ We changed the consumption parameters in a wide range in the sensitivity analysis and increased the overall uncertainty of the model. Second, we could not find a sufficiently large data set containing 24‐hour urine sodium measurements at the individual level, and the dietary recall data we relied on were subject to the limitations of survey studies, which may provide conservative results of expanded interventions benefit.^46^ Finally, the RRs for assessing risk factors can vary across subpopulations and locations, but specific RRs are not available for each province and age group^26^; moreover, the prevalence and mortality of GCa are country‐level data, which could attenuate disparities in DALY burden across provinces.

Future studies may consider cost and examined the cost‐effectiveness of different restriction strategies. Further studies should estimate the socioeconomic equity in the health of existing and potential China dietary salt reduction interventions.

## CONFLICTS OF INTEREST

The authors declare no conflicts of interest.

## ETHICS STATEMENT

Ethical approval was not required for this study, as it is an analysis of previously collected data. Ethical approval for each survey was obtained by the Health Survey for CHNS team.

## AUTHOR CONTRIBUTIONS

**Yiping Zeng**: Conceptualization, data curation, formal analysis, validation, visualization, writing—original draft. **Zeshui Xu**: Writing—review and editing, supervision. **Yu Rao**: Writing—review and editing. All authors have read and approved the final version of the manuscript. The corresponding author had full access to all of the data in this study and takes complete responsibility for the integrity of the data and the accuracy of the data analysis.

## Supporting information

Supporting information.
