# Supplementary material for: Predicting the effectiveness of interventions on population‐level sodium reduction: A simulation modeling study
Source: Health Sci Rep. 2022 Mar 7;5(2):e540. doi: 10.1002/hsr2.540 (PMC8900977; doi:10.1002/hsr2.540)
Supplement: Supplementary file 1 — Supporting information. [file HSR2-5-e540-s001.docx]

# **SUPPLEMENTAL APPENDIX**

**
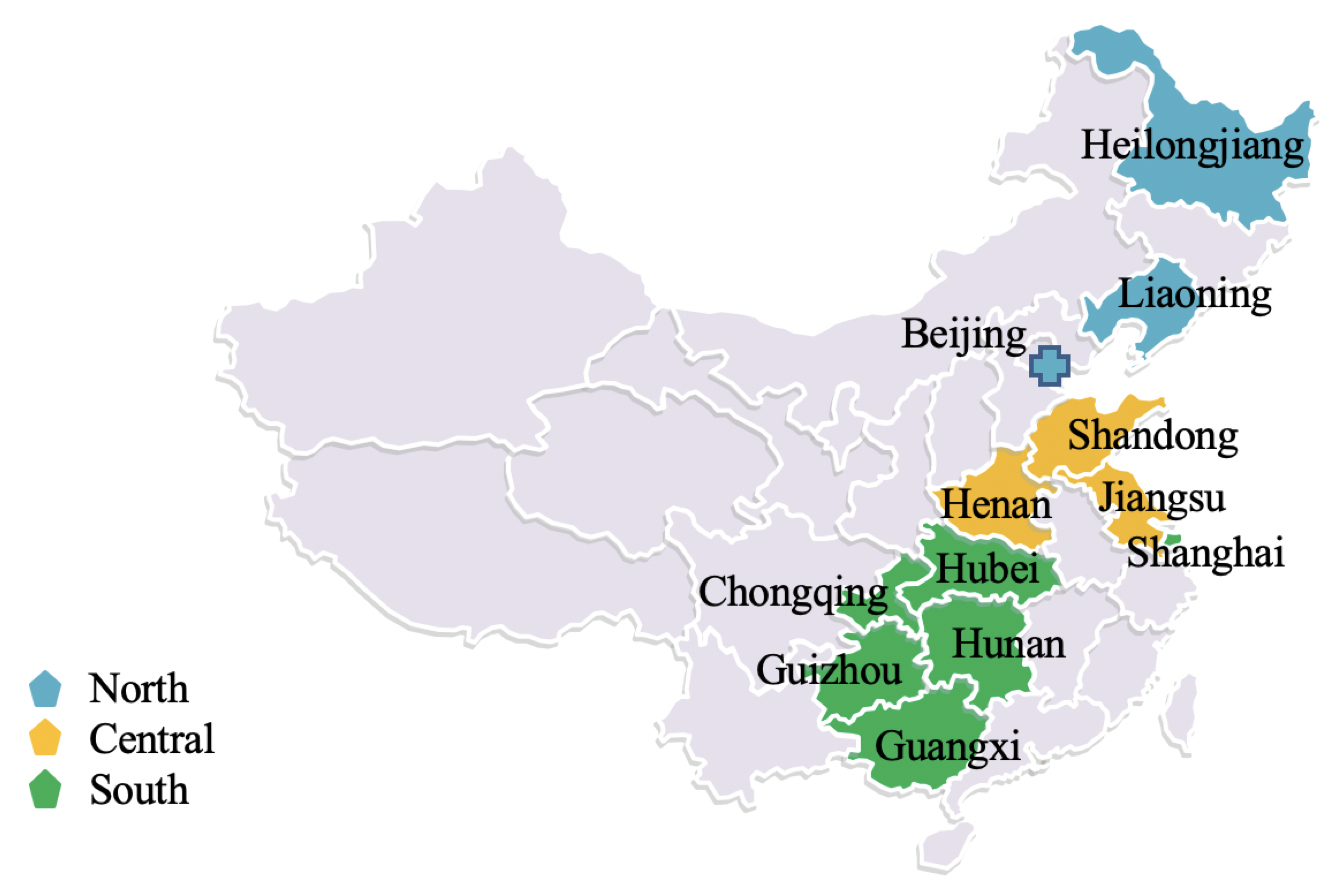
**

**S1 Fig. Modeled provinces.**

Modeled provinces are shown in color corresponding to their region.

**S1 Text: Model structure**

***Model population***

Our model evaluated all provinces subject to data availability (12 provinces in CHNS). By 2011 the provinces included in the CHNS sample constituted 47% of China’s population (according to the 2010 census). The model aggregated results from individual provinces to compute results nationally and for three regions, which include the North (Beijing, Heilongjiang, and Liaoning), the Central (Henan, Jiangsu, and Shandong), and the South (Chongqing, Hunan, Hubei, Guizhou, Guangxi, and Shanghai). Our method of separating the 12 provinces into 3 regions based on geographic locations associated with major dietary differences is consistent with the classification of CHNS. See S1 Fig for a map of modelled provinces. We synthesize information from the National Bureau of Statistics (NBS) regarding China population structure by age, sex and province (the sixth census in 2010) and the Health Survey for China (CHNS 2004-2011) regarding exposure to GCa-associated risk factors (see below) to generate a close-to-reality synthetic population. The ‘close to reality’ synthetic population ensures that the simulated synthetic individual samples are drawn from synthetic populations that are similar to the real one in terms of age, sex, province, and risk factor conditional distributions. In our model, we used the same statistical framework originally developed by Alfons et al [suppl. Ref. 1] and adapted it to make it compatible with epidemiological principles and frameworks. In general, this approach uses a nationally representative survey of the real population to generate a ‘close to reality’ synthetic population. Thus, the approach expands the survey samples (usually small) into a significantly larger synthetic population, while retaining the statistical properties and important relevance of the original survey.

In this study, the number of synthetic individuals entering the simulation was set to 10,000. Each individual was probabilistically given values of sodium consumption, morbidity risk, and mortality risk. Finally, YLDs, YLLs, and DALYs for specific cause were calculated for each person.

***Risk factors***

Excessive dietary salt intake has been associated with an increased risk of gastric cancer (GCa) [2, 3]. Studies of a high-salt diet promoting the colonization of Helicobacter pylori (a widely accepted risk factor for GCa) in the stomach by changing the viscosity of the gastric mucosal barrier is well established, although the pathophysiological mechanism linking excess salt to the increased risk of GCa are not yet well understood [4].

Exposures to carcinogens during early life can affect an individual's cancer risk by acting during crucial developmental periods and increasing cumulative mutagenic damage [5]. GCa trends in young adults not only reflect recent changes in carcinogenic exposure but could also foreshadow that the future burden of GCa might be exacerbated as younger cohorts age. Differences in the lag time of risk reversibility render the implementation of experimental studies about salt risk reversibility on GCa impossible on the ethical ground [6]. For example, consider a randomized controlled trial to study the effect of interventions to reduce salt consumption on GCa. Considering the gradual increase in the incidence of GCa in the 15-30 age group and increasing risk of excess salt consumption-induced related diseases in contemporary birth cohorts, and the mean lag time between exposure and GCa is 8 years (range 1 to 10 years), we created a microsimulation in which synthetic individuals were exposed to sodium-intake-related changes in dietary patterns until the simulation horizon is reached, or death occurs. We found that the uncertainty intervals around the model parameters were reasonable over this simulated period.

As the simulation progressed in annual circles, risk factors were updated yearly for each synthetic individual in the model. The age of each person increases by one year in each loop, and the sex remains stable. From the original surveys in the 9 provinces (including Guangxi, Guizhou, Heilongjiang, Hubei, Hunan, Henan, Jiangsu, Liaoning, and Shandong), we simulated a decline in sodium intake observed between 2004 and 2011, assuming a logarithmic decline.  In addition, for the three largest municipal cities (Beijing, Shanghai, and Chongqing) that were added in 2011, we assumed that when salt-related interventions were not implemented sodium exposure remained stable at the estimated level of 2011 until the period up to 2030.

***Model Validation***

Three sets of model results were validated for all populations, including intermediate results in 2015, one-year disease burden results, and 20-year disease burden results (calculated based on burdens of one-year and assuming continuation of secular trends without interventions). Before using our microsimulation model to estimation DALYs, we ensured that results are in good concordance with the literature values both at the country and region levels.

For intermediate results, modeled sodium intake in 2015 were compared to estimates from CHNS et al [7, 8] (Suppl. Table S4). For one-year disease burden results, modeled DALYs from GCa in 2010 were compared to estimates from the GBD (Suppl. Table S5). For 20-year disease burden results, modeled DALYs from GCa in 2010 to 2030 were compared to estimates from the GBD (Suppl. Table S6). The GBD estimates used for validation were not involving our simulation modelling projections, but was derived from Bayesian statistics model provided by GBD.

**S1 Text References**

1. Alfons A, Kraft S, Templ M, et al. Simulation of close-to-reality population data for household surveys with application to EU-SILC. Statistical Methods and Applications. 2011;20(3):383-407.
2. D’Elia L, Rossi G, Ippolito R, et al. Habitual salt intake and risk of gastric cancer: a meta-analysis of prospective studies. Clinical Nutrition. 2012;31(4):489-98.
3. Simon C, Martin O, Rapid mortality falls after risk-factor changes in populations. Lancet. 2011;378(9793):752-753.
4. Fox J, Dangler C, et al. High-salt diet induces gastric epithelial hyperplasia and parietal cell loss, and enhances Helicobacter pylori colonization in C57BL/6 mice. Cancer Research. 1999;59(19): 4823-8.
5. Clarke MA, Joshu CE, Early life exposures and adult cancer risk. Epidemiologic Reviews. 2017;39:11-27.
6. World Cancer Research Fund, American Institute for Cancer Research. Food, nutrition, physical activity, and the prevention of cancer: a global perspective. Washington, DC: WCRF/AICR; 2007.
7. Popkin B M, Du S, Zhai F, et al. Cohort profile: the China Health and Nutrition Survey-monitoring and understanding socio-economic and health change in China, 1989-2011. International Journal of Epidemiology, 2010;39(6):1435-1440.
8. Du S, Wang H, Zhang B, et al. Dietary potassium intake remains low and sodium intake remains high, and most sodium is derived from home food preparation for chinese adults, 1991-2015 trends. The Journal of Nutrition. 2020;150(5):1230-1239.

| **S1** **Table.**  Characteristics of individuals with data on salt consumption (*n* = 22,887), China Health and Nutrition Survey 2004-2011 | |
| --- | --- |
| **Characteristic** | **Weighted %**  **(unweighted n) ^1^** |
| *Sex* |  |
| Female  Male | 49.1 (11,159)   50.9 (11,728) |
| *Age* |  |
| 0-19 y  20-39 y  40-59 y     60+ y | 23.1 (4,837)  32.3 (5,325)  30.3 (7,695)  14.3 (5,030) |
| *Region* **^2^** |  |
| North  Central  South | 16.3 (4,914)  42.9 (6,499)  40.8 (11,474) |
| Meet WHO recommendation for the tolerable upper sodium intake (≤ 2g/d) **^3^** | 14.8 (3,403) |
| **^1^** Percent accounts for sampling design with survey weights rescaled according to Sixth Census of the National Bureau of Statistics of China in 2010.  **^2^** Provinces included: North (Beijing, Heilongjiang, and Liaoning), Central (Henan, Jiangsu, and Shandong), and South (Chongqing, Hunan, Hubei, Guizhou, Guangxi, and Shanghai).  **^3^** Participants were classified as meeting the WHO recommendation [30] if they self-reported consuming ≤ 2g of sodium per day. | |

| **S2 Table.**  Consumption of sodium among individuals, China Health and Nutrition Survey 2004-2011 | | |
| --- | --- | --- |
| **Characteristic** | **Meet WHO recommendation ^12^** | **Sodium intake (g/d) ^3^** |
| ***Mean*** | 14.8 (13.9-15.6) | 4.7 ± 2.3 |
| ***North*** | 16.4 (14.2-19.3) | 4.8 ± 2.4 |
| Beijing  Heilongjiang Liaoning | 24.1 (24.1-24.1)  12.5 (12.1-13.3)  14.6 (14.1-15.1) | 3.1 ± 2.3  4.9 ± 2.2  6.3 ± 2.6 |
| ***Central*** | 13.6 (12.8-14.2) | 5.5 ± 2.5 |
| Henan  Jiangsu  Shandong | 13.4 (12.7-14.3)  13.6 (13.1-14.2)  13.9 (13.3-14.7) | 7.5 ± 2.2  6.8 ± 2.2  7.4 ± 2.3 |
| ***South*** | 14.8 (14.1-15.5) | 4.7 ± 2.2 |
| Chongqing  Hunan  Hubei  Guizhou  Guangxi  Shanghai | 20.5 (20.5-20.5)  10.3 (9.4-10.8)  8.2 (7.7-8.5)  9.3 (8.4-9.9)  12.1 (11.7-12.5)  37.8 (37.8-37.8) | 3.9 ± 2.0  4.4 ± 2.1  7.5 ± 2.1  4.2 ± 2.2  4.2 ± 2.1  3.1 ± 2.0 |
| ***sex*** |  |  |
| Female  Male | 15.0 (14.4-15.6)  14.6 (14.1-15.2) | 4.6 ± 1.9  4.7 ± 2.5 |
| ***Age*** |  |  |
| 0-19 y  20-39 y  40-59 y     60+ y | 14.5 (13.9-15.1)  15.1 (14.7-15.5)  13.8 (13.1-14.5)  16.4 (15.9-16.8) | 4.1 ± 2.1  4.6 ± 2.1  5.6 ± 2.3  4.5 ± 2.2 |
| **^1^** Values are % (95% CrI) and take into account the sampling design.  **^2^** Participants were classified as meeting the WHO recommendation [30] if they self-reported consuming ≤ 2g of sodium per day.  **^3^** Value are given as means ± SDs. | | |

| **S3 Table.**  Distributions used in probabilistic sensitivity analysis. | |
| --- | --- |
| **Parameter** | **Probability Distributions** |
| Sodium intake | Log-Normal |
| GCa prevalence rate | Beta |
| Relative risk of GCa | Gamma |
| GCa morality rate | Gamma |

| **S4 Table.**  Validation of sodium dietary intake in 2015.  Results are shown as percent changes from literature values to model results. | |
| --- | --- |
| **Region** | **Sodium intake (%)** |
| National | 0.6 |
| North | 1.2 |
| Central | 0.1 |
| South | 7.7 |

| **S5 Table.**  Validation of DALYs from GCa in 2010.  Results are shown as percent changes from literature values to model results. | |
| --- | --- |
| **Region** | **Gastric Cancer (%)** |
| National | 1.9 |
| North | 1.2 |
| Central | -0.3 |
| South | 3.4 |

| **S6 Table.**  Validation of DALYs from GCa from 2010 to 2030.  Results are shown as percent changes from literature values to model results. | |
| --- | --- |
| **Region** | **Gastric Cancer (%)** |
| National | -0.8 |
| North | -1.3 |
| Central | -0.4 |
| South | 0.5 |

| **S7 Table.**  Results of probabilistic sensitivity analysis for DALY burdens due to sodium intake changes from 2010 to 2030. | | |
| --- | --- | --- |
| **Region** | **Burden**  **(10^6^ DALYs)** | **95% Credible Interval**  **(10^6^ DALYs)** |
| National | 20.2 | (8.1, 33.4) |
| North | 5.9 | (4.6, 7.2) |
| Central | 9.8 | (8.9, 10.2) |
| South | 4.5 | (2.6, 6.7) |

| **S8 Table.**  Sensitivity analysis with sodium intake distributions.  Sodium intake distributions were assumed to be lognormal.  The model was run 10,000 times with 10,000 people while sampling from distributions reflecting uncertainty in inputs. | | |
| --- | --- | --- |
| **Region** | **Burden**  **(10^6^ DALYs)** | **95% Credible Interval**  **(10^6^ DALYs)** |
| National | 19.8 | (8.7, 31.9) |
| North | 6.2 | (4.5, 7.1) |
| Central | 8.7 | (8.0, 9.4) |
| South | 4.9 | (3.0, 7.0) |

| **S9 Table.**  Sensitivity analysis with constant GCa prevalence rates.  GCa prevalence rate in 2010 was assumed to continue over the model period.  The model was run 10,000 times with 10,000 people while sampling from distributions reflecting uncertainty in inputs. | | |
| --- | --- | --- |
| **Region** | **Burden**  **(10^6^ DALYs)** | **95% Credible Interval**  **(10^6^ DALYs)** |
| National | 21.4 | (8.5, 34.3) |
| North | 6.0 | (4.7, 7.6) |
| Central | 9.9 | （8.7, 10.8) |
| South | 5.5 | (3.5, 7.5) |

| **S10 Table.**  Projected raw and per capita DALY burdens due to excess salt consumption from 2010 to 2030 in the scenario without interventions.  Sodium intake in 2015 are also shown. | | | |
| --- | --- | --- | --- |
| **Province** | **Burden**  **(10^6^ DALYs)** | **Burden per capita**  **(DALYs/10^3^ ppl)** | **Sodium Intake**  **(mg/capita/day)** |
| Beijing | 1.7 | 7.1 | 2734 |
| Liaoning | 2.1 | 10.5 | 4517 |
| Heilongjiang | 2.3 | 9.4 | 5912 |
| Shanghai | 0.9 | 2.8 | 2705 |
| Jiangsu | 2.4 | 8.7 | 6351 |
| Shandong | 2.9 | 15.8 | 6953 |
| Henan | 2.5 | 13.4 | 7124 |
| Hubei | 1.5 | 4.6 | 7069 |
| Hunan | 1.1 | 5.3 | 4018 |
| Guangxi | 1.3 | 2.8 | 3843 |
| Guizhou | 0.7 | 5.7 | 3816 |
| Chongqing | 0.2 | 2.5 | 3512 |
